# Current relevance of hypoxia in head and neck cancer

**DOI:** 10.18632/oncotarget.9549

**Published:** 2016-05-21

**Authors:** Marius G. Bredell, Jutta Ernst, Ilhem El-Kochairi, Yuliya Dahlem, Kristian Ikenberg, Desiree M. Schumann

**Affiliations:** ^1^ Department of Cranio-, Maxillofacial and Oral Surgery, University Hospital Zürich, Zürich, Switzerland; ^2^ Department of Pathology, University Hospital of Zürich, Zürich, Switzerland

**Keywords:** hypoxia, head and neck cancer, oral cancer, biomarkers

## Abstract

Head and Neck cancer (HNC) is a complex mix of cancers and one of the more common cancers with a relatively poor prognosis. One of the factors that may assist us in predicting survival and allow us to adjust our treatment strategies is the presence of tumor hypoxia. In this overview we aim to evaluate the current evidence and potential clinical relevance of tumor hypoxia in head and neck cancer according to an extensive search of current literature.

An abundance of evidence and often contradictory evidence is found in the literature. Even the contradictory evidence and comparisons are difficult to judge as criteria and methodologies differ greatly, furthermore few prospective observational studies exist for verification of the pre-clinical studies. Despite these discrepancies there is clear evidence of associations between prognosis and poor tumor oxygenation biomarkers such as HIF-1α, GLUT-1 and lactate, though these associations are not exclusive. The use of genetic markers is expanding and will probably lead to significantly more and complex evidence. The lack of oxygenation in head and neck tumors is of paramount importance for the prediction of treatment outcomes and prognosis. Despite the wide array of conflicting evidence, the drive towards non-invasive prediction of tumor hypoxia should continue.

## INTRODUCTION

Head and neck cancer (HNC) constitutes 5.1% (> 633 000) of all new cancers and relates to 4.8% of all cancer deaths annually worldwide with Head and Neck Squamous Cell Carcinomas (HNSCC) contributing by far the largest number [1]. Major risk factors are smoking (smokers are ten times more likely to develop HNSCC than non-smokers), alcohol abuse and HPV infection [2]. The main sites for HNSCC are the larynx, pharynx and oral cavity [1]. HNSCC is common only in its general anatomic localization as it is a diverse disease with regards to etiology, presentation, response to treatment and prognosis. Despite this diversity, there are some common features that may lead to local and regional recurrence and predict disease-specific survival. One of these features is the presence of hypoxia in the tumor [3].

Since hypoxic tumors show a poorer response to surgery and radiotherapy than non-hypoxic tumors, prior therapy knowledge on the presence and extent of the hypoxia is needed [4, 5]. Currently, except for in-dwelling catheters, adequate information regarding the extent of the tumor hypoxia can only be gathered after tumor resection and histological processing. A wide array of tumor hypoxia biomarkers has been identified, however, to our knowledge no pre-excision predictive parameters are available. Except for the radio-oncology field where hypoxia predictive markers are now being implemented in some clinics, evidence is lacking regarding modifications that may be undertaken to optimize treatment [6-8].

The goal of this paper is to demonstrate the complexity, summarise and discuss the current literature evidence and potential clinical relevance of tumor hypoxia and its biomarkers in HNC. A PubMed and Medline search was performed using the keywords tumor hypoxia, pimonidazole, biomarkers, HIF-1alpha, CA-IX, Nitric oxide, smoking, anemia, VEGF, lactate, miRNA, head and neck cancer, and head and neck squamous cell carcinoma either alone or in combination. Articles were screened according to their level of clinical and scientific evidence as well as clinical relevance to the topic.

## WHAT IS TUMOR HYPOXIA?

Tumor hypoxia can be defined as a low oxygen tension (pO_2_) in the tumor compared to the surrounding tissue of pO_2_ ≤ 2.5 mmHg, but is most commonly defined as a pO_2_ ≤ 10 mmHg [9]. A functional definition may be more appropriate in such an individualized and complex environment and therefore, at our current level of knowledge, tumor hypoxia starts when Hypoxia-inducible Factors (HIF)-subunits become stabilized due to limited oxygen availability compared to oxygen demand. More than 50% of solid tumors display heterogenous hypoxic areas irrespective of their size and histological characteristics [10-12]. Oxygen content within tissues is dependent on a number of complex factors such as the physical presence of oxygen, the programming of tumor and stroma cells whether to utilize the available oxygen, vasculature, perfusion and diffusion distance that may be influenced by vascular density and edema, as well as systemic factors such as anemia or chronic obstructive lung disease.

## GENERAL CONDITIONS INFLUENCING TUMOR HYPOXIA

### Role of vasculature/microcirculation in tumor hypoxia

The vasculature in tumors originate from host vessels and neovascularisation due to tumor angiogenesis factors [13]. If the incorporated vasculature is insufficient for the tumor mass, reduction in oxygen exchange will develop. This hypoxic state will stimulate reperfusion via neovascularisation in an attempt to restore the blood flow and improve the oxygen supply via a variety of metabolic pathways of which the HIF-1 alpha pathway is central [14, 15]. This state of poor vascularity in terms of density, quality of blood flow, increased diffusion distance and permeability may also implicate poorer drug delivery and treatment response [16, 17].

**Figure 1:**
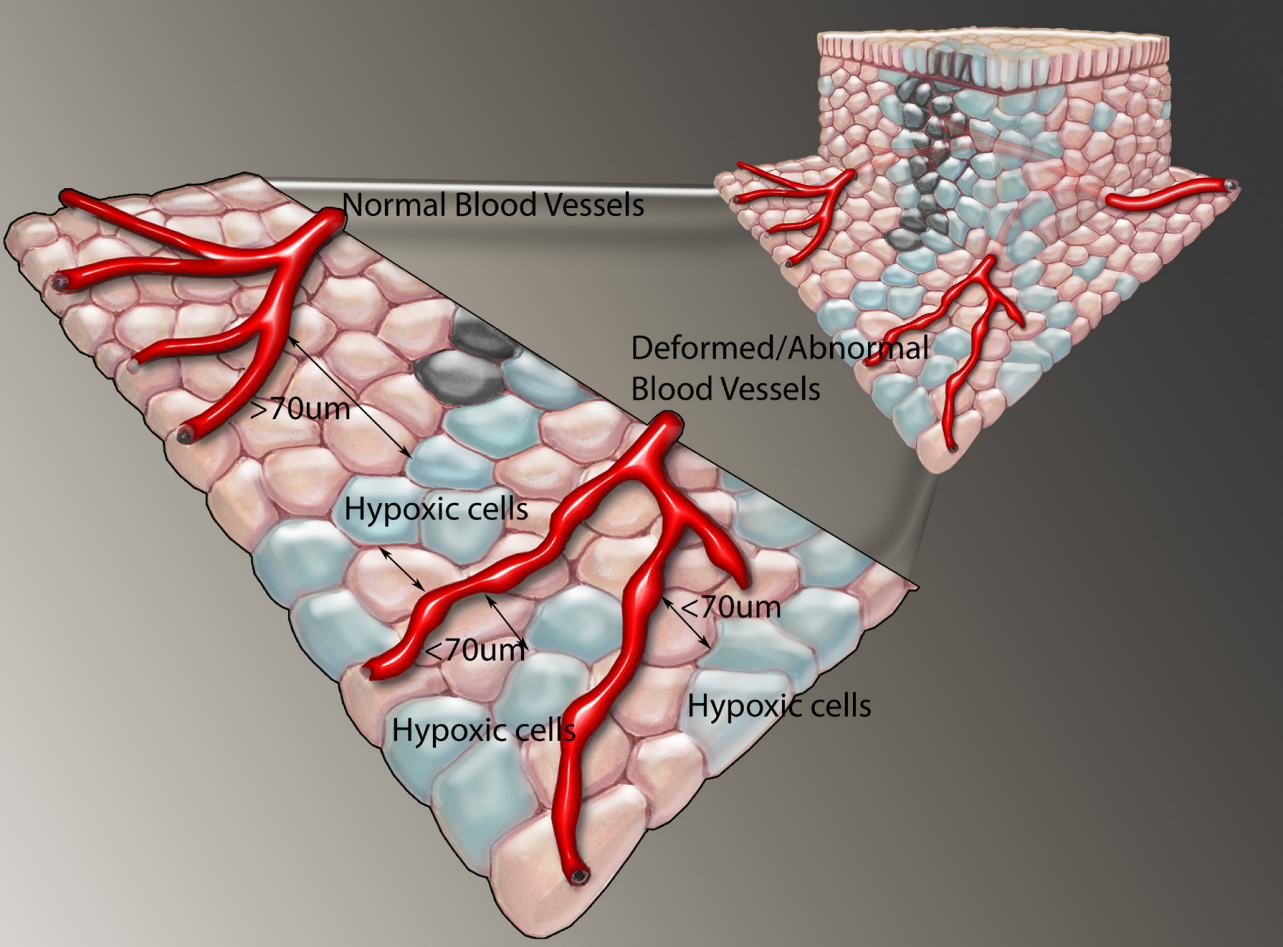
Tumor showing hypoxic cells beyond the oxygen diffusion limit of 70 μm and close to newly formed tortuous, haphazard blood vessels that have sluggish perfusion Functional venous drainage, preventing edema is also important, but not demonstrated here.

The neovascularisation induced vessels are often tortuous and haphazard resulting in a sluggish perfusion, increased vessel permeability and distance from intact blood vessels [3, 18] (Figure 1). Therefore, a highly vascularised tumor is not necessarily indicative of a highly oxygenated tumor [13]. This vascular-dependent relative hypoxic state does not necessarily lead to an anaerobic metabolic state of the tumor cells (Warburg effect).

Two subtypes of hypoxia have been described. Acute hypoxia is defined as a local disturbance in perfusion. The disturbance can be caused by the loss of microvascular blood flow or by strong variations in red blood cell fluxes which results in decreased microvascular oxygen supply to the tumor area due to the tortuous and aberrant vasculature and may be a transient phenomenon [19, 20]. Upon revascularization with well-oxygenated blood, free oxygen radicals are released causing further tissue damage and even necrosis. When tumor growth is greater than neo-vascularisation, perivascular necrosis develops at a distance of 130-150μm from nutrient vessels and cells further away than 70 μm from the vessels already show hypoxic tension indicating the oxygen diffusion limit (Figure 1). This phenomenon is called chronic or diffusion-limited hypoxia and can activate stress-response genes and is typical of a re-vascularisation or re-oxygenation injury, explaining central necrosis, often found in fast growing tumors. This central necrosis is at least partially the cumulative end result of both acute and chronic hypoxia in the tumor microenvironment [21]. Especially in initial stages, cancer cells may also need other growth factors, and they will be selected by necrosis until they gain a mutation that renders them growth factor independent. Generating a universal definition of hypoxia may thus be difficult and probably needs to sub-defined depending on the particular measurement assay which may be physical or a genetically or local environment dictated metabolic measurement. Important also is to consider non-inherent tumor factors, like anemia, smoking, decreased lung function or post radiation related poor perfusion in HNC patients that may influence tumor hypoxia. Decreased blood flow has a negative effect on the oxygen delivery to a tumor area and this is often termed hypoxia, but strictly defined, the term ischemia should be used for this phenomenon. Hypoxemia is defined as less oxygen in the blood due to decreased hemoglobin-oxygen saturation.

### Anemia and tumor hypoxia

Anemia plays a role in tumor hypoxia [13]; it is defined as a hemoglobin (Hb) level less than 12.0 g/dl in females and less than 13.8 g/dl in males, and has long been known as a prognostic factor in HNC [3]. Post-operative acute anemia (Hb<12 g/dl) has been described as an independent prognostic factor for local recurrence-free survival [5, 22, 23]. Nordsmark et al. [24] and Nordsmark [25] found a correlation between median pO_2_ levels (9 mmHg; range 0-62 mmHg) and Hb levels but when they subcategorized into pO_2_<2.5 mmHg and pO_2_<5 mmHg, they found no correlation between pO2_2_ and Hb.

Pre-operative anemia plays a significant role in overall survival [23]. Low Hb levels may impair survival by impairing tissue and possibly tumor oxygenation causing hypoxia and thereby reducing the effectiveness of chemotherapy and radiotherapy [26, 27]. Compounding the complexity of the matter is evidence that blood transfusion may lead to a worse prognosis [28]. Bhide et al. [29] and Hoff et al. [30] found that blood transfusion before and during radiotherapy in HNC patients had no effect on survival and may in fact be deleterious. This paradox may be explained by the leakage of endothelial growth factors from the aging red blood cells that may encourage tumor growth and have a deleterious effect on immune regulation. This evidence should encourage us to move away from the common notion of transfusing patients with an Hb < 10 g/dl or a hematocrit (Hct) < 30%. Vaupel et al [31] found that an Hb level between 12 and 14 g/dl was optimal for tumor oxygenation even though no correlation has been found between Hb level and pO_2_ [32, 33].

Erythropoietin (EPO) is a glycoprotein that regulates erythrocyte production by stimulating growth, preventing apoptosis and inducing differentiation of red blood cell precursors. Increasing the Hb levels with EPO has led to contradictory results. Henke et al. [34] showed a decrease in recurrence in pelvic malignancies in patients administered erythropoietin alfa and iron compared to patients who received iron only. It was later shown that EPO treatment during radiotherapy may have a detrimental effect on loco-regional progression-free survival and overall survival in HNC patients despite an increased Hb [35]. This negative effect of EPO could be due to the fact that the Hb levels were increased to around 14.8 g/dl which falls outside the beneficial range (12 g/dl to 14 g/dl) suggested by Vaupel et al [31]. This argument is controversial because in the experiments performed by Glaser et al [36], two groups had a starting Hb level > 14.5 g/dl and these patients had a good overall response to neo-adjuvant chemotherapy. Neither Henke et al. [35] nor Glaser et al. [36] investigated the effects of EPO administration on tumor hypoxia. *In vivo*, EPO appears to have no effect on tumor growth or radiotherapy efficacy [37, 38]. Furthermore, no correlation has been found between Hb and tumor hypoxia markers such as HIF-1alpha, HIF-2alpha, and Carbonic Anhydrase-IX (CA-IX) [39, 40], however in a cervical cancer cohort anemia did correlate with HIF-1alpha expression [27].

Total Hb levels cannot be used to indirectly determine oxygen delivery to tissue as it does not take into account blood flow, Hb saturation, oxygen-Hb dissociation, or the quality of the Hb [41].

Although no overwhelming evidence, anemia has been shown to interrelate anemia and tumor hypoxia, anemia remains an important factor in patient survival. There is, however, evidence that implicates carboxyhemoglobin in tumor hypoxia.

### Smoking and tumor hypoxia

Smoking is one of the main risk factors for HNSCC and the main source of carbon monoxide (CO). Patients who continue smoking during radiation have a worse prognosis than non-smokers [42, 43]. Carboxyhemoglobin is defined as Hb-bound CO [44]. Hb has an approximately 200-280 fold higher affinity for CO than for oxygen, therefore low amounts of CO will impact on the oxygen-hemoglobin dissociation curve by shifting it left, resulting in diminished tumor blood perfusion as well as the amount of oxygen bound to Hb [44]. Increased HbCO in HNC patients who smoke, results in a decreased oxygen unloading capacity [41]. This means that most of the oxygen that enters a tumor subsequently leaves the tumor in venous blood outflow. A rise of HbCo to 12 % (compared to the average of 4.6% in smokers) results in a 25 % reduction in oxygen available to the tumor [41]. CO causes a 50 % reduction in blood flow to tumors and a 50 % increase in tumor hypoxia in mice [45].

Cigarette smoke also contains nicotine which impairs wound healing by vasoconstriction thus decreasing blood flow to the wound [46]. Nicotine and its derivatives play a role in tumor progression and metastasis by increasing oxidative stress and activating NF-kappaB and other proliferation signaling pathways [47]. Nicotine promotes proliferation of nasopharyngeal carcinoma cells by inducing HIF-1alpha and vascular endothelial growth factor (VEGF) and inhibiting pigment epithelium-derived factor (PEDF), a known anti-angiogenic, anti-tumorigenic protein [48].

### Alcohol and tumor hypoxia

**Figure 2:**
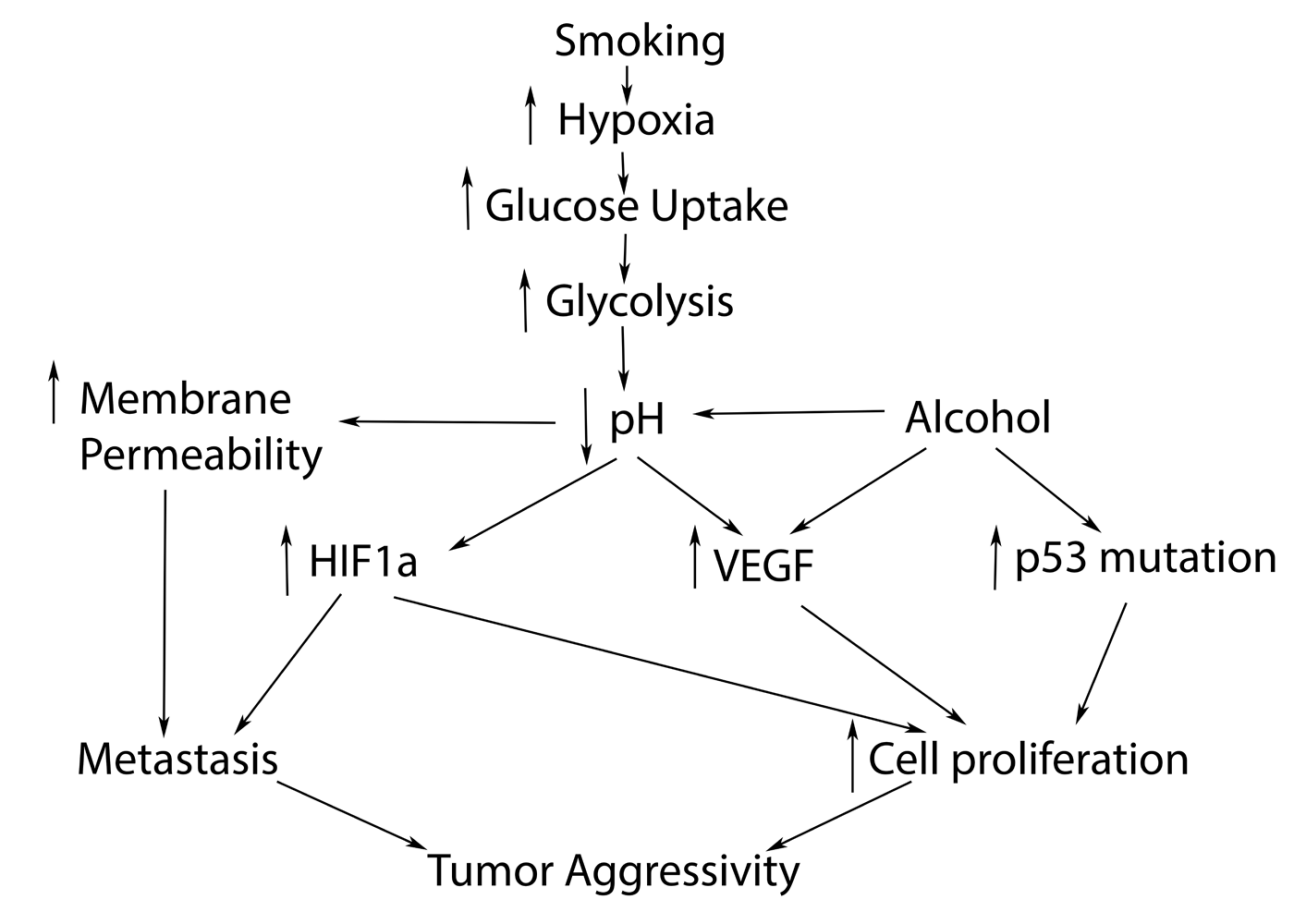
Hypoxic pathway possibly relates to smoking, leading to increased tumor aggressivity and poorer prognosis in HNC

Alcohol and more so, alcohol and smoking combined, are major risk factors for HNSCC (Figure 2) as alcohol may act as a solvent for cigarette smoke. Although no direct correlations have been made between alcohol and tumor hypoxia, alcohol increases HIF-1alpha via oxidative stress in a rat model for alcoholic liver disease [49]. Alcohol is metabolized to acetaldehyde, a known carcinogen, by alcohol dehydrogenase (ADH) situated in the liver, gastric mucosa, oesophageal mucosa and oral mucosa [50]. Most of the harmful effects of alcohol are mediated via acetaldehyde through DNA damage, but alcohol can increase mucosal permeability and cause changes in mucosal morphology [50]. The relationship between alcohol consumption and tumor hypoxia in HNC has not been investigated thus far. However, baboons fed alcohol chronically showed decreased oxygen consumption in the liver accompanied by increased acetaldehyde concentrations and impaired mitochondrial function [51]. It is therefore possible that alcohol also plays a role in tumor hypoxia in HNSCC. Not all HNSCC patients drink alcohol and/or smoke tobacco which brings our focus to the human papillomavirus.

### Human Papillomavirus (HPV) and tumor hypoxia

HPV causes up to 70 % of oropharyngeal cancers but less oral cancers. Contradicting results exist pertaining to the effect of HPV on HIF-1alpha. HPV-16 induces HIF-1alpha expression *in vitro* and *ex vivo* in head and neck tumor specimens [52-54]. Hong et al. [55] however, found no relationship between HPV-status and HIF-1alpha expression in patients with oropharyngeal cancer but patients with the worst prognosis tended to be those who were HPV-negative and HIF-1alpha positive.

HPV-positive tumors grow faster and show aggressive regional metastasis, yet the patients have a better prognosis and a better response to radio- and chemotherapy [56]. Comparatively, tumor hypoxia has also been linked to increased metastasis, but has a worse prognosis and a worse response to radio- and chemotherapy. Rodolico et al. [54] concluded that the increase in HIF-1alpha in HPV-positive tumors was oxygen-independent since the HIF-1alpha immune reactivity also occurred close to blood vessels, however Janssen et al. [57] also found that this immunoreactivity did not correlate with the pimonidazole staining. Trinkaus et al. [58] could not find an association between p16-positive patients with locally advanced head and neck tumors and hypoxic imaging using (18F)-misonidazole positron emission tomography, equally Mortensen et al. [59] could not demonstrate this by use of FAZA-PET in the DAHANCA trial. One would expect a less pronounced hypoxia association as HPV-positive patients with oropharyngeal cancer mostly have a more favorable prognosis, however the role of HPV in tumor hypoxia still has to be fully explored. Comprehensive gene profiling of HNSCC has been performed, highlighting the different pathways of HPV and non HPV induced HNSCC, but not yet covering all hypoxia pathways [60]. Comprehensive discussion of HPV and its relation to HNSCC is not within the scope of this paper.

HPV causes HNSCC in a different way with, non-mutated p53 and producing E6 and E7 oncogenes with its own unique way to manipulate the immunological system. Clearly immunity is becoming a focal point of cancer research.

### Immune system and tumor hypoxia

Inflammation appears to play a central role in the development of cancer and as far back as 1863, Virchow postulated that a process of chronic irritation or irritants may lead to injury and an inflammatory process that will enhance cell proliferation and development of cancer, including oral cancer [61, 62]. Cigarette smoke also affects the immune system by impairing immunity in the oral cavity and promoting gingival and periodontal disease and oral cancer [63]. Cigarette smoke contains bacterial lipopolysaccharides, reactive oxygen species and other reactive compounds that induce chronic inflammation in the oral mucosa and modify host responses to exogenous antigens [63]. Viral infections and especially HPV in HNSCC may play a role in immunosuppression by infecting pluripotent stem cells, resulting in oncogenic changes that are replicated in the background of a poorly responsive immune system [10, 62, 64]. The expressions of the viral oncogenes E6 and E7 are important in the process of changing stem cells because they lead to the inactivation of one of the immune defense mechanisms, the tumor suppressor genes p53 and pRb [65]. A biological explanation for the improved prognosis of HPV-positive versus HPV-negative cancer patients has not been established. Bose et al. [10] hypothesized that p53 and pRb remain intact in HPV-positive cases whereas they are mutated in HPV-negative cases.

Multiple pathways for the initiation and later association and reactive changes of inflammation and tumor development have been described. Loss of cellular senescence and development of cancer stem cells may be propagated by stress factors like inflammation and hypoxia [66]. Chronic inflammation associated with poor oral hygiene and Lichen Planus (an autoimmune disease) and oral cancer has been shown in more than 1% of afflicted patients [62]. In early premalignant lesions, inflammation-induced neovascularisation has been observed in the stromal component of verrucous hyperplasia and hyperkeratosis [67]. The composition of cytokine/chemokines, liberated from monocytes at a site of inflammation and which are important in the development of a chronic state of disease, can broadly be divided into pro-inflammatory and anti-inflammatory cytokines. One of these cytokines is tumor necrosis factor-α (TNF-α), a pro-inflammatory cytokine that has both a positive and negative effect on inflammation and has a controlling effect on pro-inflammatory cell populations. Interleukins 1 and 6 are associated with tumor metastasis and interleukins 8 and 12 have been associated with inflammation and cancer [61, 62].

The role of inflammation, hypoxia, lactate, glucose metabolism and angiogenesis in oncogenesis is highlighted by various associations like CXCL12 that may play a role in the immune response by activating CD8 and T cells in established HNSCC [62, 68]. CXCR4 and CXCL12, both also described as HIF targets, are highly expressed in HNSCC and correlate with poor prognostic outcome due to increased metastasis and increased resistance to therapy [69]. Glucose and its possible role in tumor hypoxia, along with diabetes is described later in this manuscript.

In a study by Dumitru et al. [70] an association between AHNAK/Desmoyokin, a giant protein associated with poorer immune defense and an increased migration inhibitory factor (MIF, also a HIF target), and increased level of neutrophil tumor infiltration was established in laryngeal cancer patients with poor prognosis. Factors released by neutrophils enhance tumor migration in a feedback manner [70]. The role of the inflammatory process and its controlling proteins and prescribing gene pool is intricate and new discoveries slowly enhance our understanding.

Up to now we have described the interplay between the tumor environment and tumor hypoxia. Pimonidazole, an exogenous biomarker for tumor hypoxia, has thus far been the gold standard. Endogenous biomarkers such as LDH-5, HIF-1α, CA-IX, GLUT-1 and MCT4 may show some clinical relevance [8, 57, 71-73].

## ENDOGENOUS BIOMARKERS IN TUMOR HYPOXIA

It is important to recognize that most biopsy specimens are adequate for diagnosis of malignancy, but may not be a representative metabolic sample of the tumor. This is because: 1. Biopsy specimens are taken from variable, mostly peripheral parts of the tumor that may represent different states of perfusion [10]; 2. Tumors often show heterogenous metabolic activity and large tumors display areas of acute and chronic hypoxia that may not be represented by a single or even multiple biopsies [25]; 3. Tumor hypoxia is a dynamic process and tumors may display signs of hypoxia earlier or later in their development; and 4. Hypoxia is not a size-dependent factor and shows a poor relationship with other clinicopathological factors like differentiation and lymph node metastasis. Despite the above, a significant relationship to prognosis exists, demonstrating poor sensitivity, specificity and a poor understanding and interrelationship of our current prognostic predictive methods [3, 15].

Janssen et al. [3] raised interesting questions regarding the endogenous markers for hypoxia – i.e. is the marker only dependent on hypoxia for its expression or do other factors play a modulating role? Is the marker expressed (under hypoxia) in all tumor types? Is the marker expressed under acute and chronic hypoxia i.e. what is the time scale for upregulation after hypoxia induction? With these questions in mind, we discuss the more common biomarkers used for tumor hypoxia.

### HIF and tumor hypoxia

**Figure 3:**
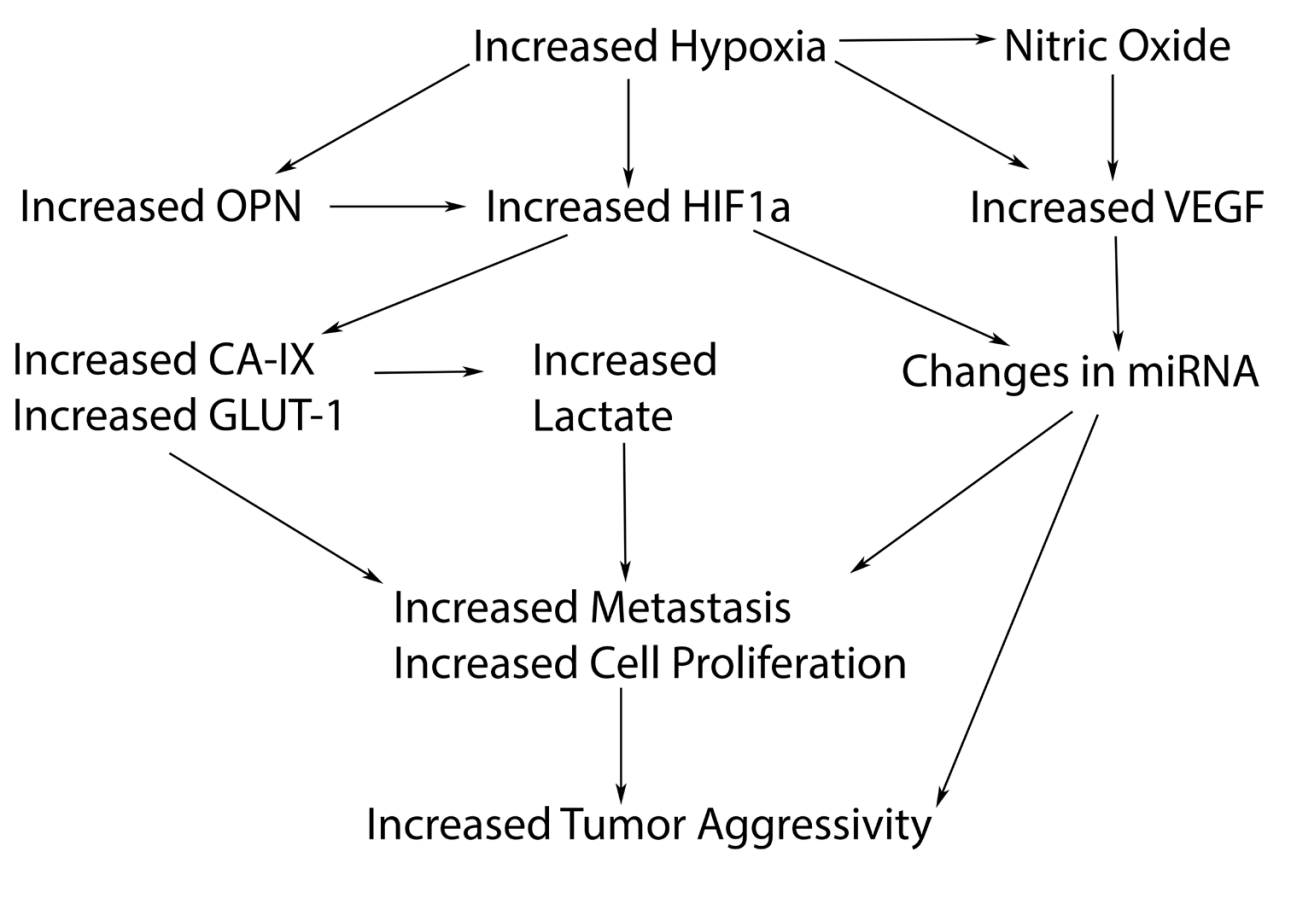
Hypoxic related biomarkers that may play a role in the hypoxic pathway leading to increased tumor aggressivity

HIF form a family of heterodimeric transcription factors that are upregulated in response to hypoxia and regulate hypoxia-driven changes in tumor cells. HIF-1alpha is a member of this family and is often used as a marker for tumor hypoxia. The understanding thus far has been that in normoxic conditions, HIF-1alpha subunits are degraded whereas in hypoxia, the degradation is inhibited when the enzymes that modify HIF become inactive. HIF-1alpha then binds to hypoxia response elements and regulates changes in expression of VEGF, CA-IX, glucose transporter (GLUT-1), EPO, plasminogen activator inhibitor-1 (PAI-1) and others (Figure 3). In recent years it has been shown that HIF-1alpha expression is not only regulated by hypoxia but by nitric oxide [74-76], reactive oxygen species [76, 77], cytokines and growth factors such as TGF-beta [78, 79] and insulin [80], factors not necessarily associated with tumor hypoxia. HIF-1alpha is activated at physiological pO_2_ in a mitogen activated protein kinase (MAPK)-dependent manner and is needed for proliferation various cancer cell lines and in normal human keratinocyte cells [81]. Janssen et al. [57] found no correlation between pimonidazole and HIF-1alpha staining and concluded that HIF-1alpha is not a suitable marker for chronic hypoxia. It would appear that HIF-1alpha is only transiently induced and then undergoes (partial) feedback inhibition [82]. Pimonidazole staining increased with distance from the blood vessels which corroborated the findings of Wijffels et al. [83]. Furthermore, Joshi et al. [84] recently found that hypoxia alone is not sufficient to stabilize HIF-1alpha and that HIF-1alpha can be degraded during hypoxia by the 26 S proteasome via the E3 ligase MDM2 [84].

Diabetes is recognized as a risk factor for oral cancer [85]. In an epidemiological study in Hungary, 24.3 % of the patients who had malignant oral lesions also had impaired glucose metabolism [86]. A higher percentage of type 2 diabetic patients, who are characterized by increased circulating insulin due to insulin resistance, had malignant lesions compared to type 1 diabetic patients. Cholesterol and insulin (both of which are elevated in Type 2 diabetes) increase HIF-1alpha. Although the effects of HIF-1alpha, along with VEGF are not unique to hypoxia, they are of significant therapeutic interest.

## VASCULAR ENDOTHELIAL GROWTH FACTOR (VEGF) AND TUMOR HYPOXIA

VEGF is a hypoxia-responsive gene and is a key player in the development of tumor vascularisation [87]. Increased VEGF production by tumor cells is associated with poor prognosis, nodal metastasis, clinical stage and low survival in HNSCC [88]. It is upregulated in decreasing concentrations of oxygen *in vitro* and *in vivo* [89-91] in HNSCC via Jun N terminal kinase (JNK-1) and p38 kinase which are stress activated protein kinases [92]. To further complicate matters, VEGF is also regulated by many other factors in normoxic conditions [93].

**Figure 4:**
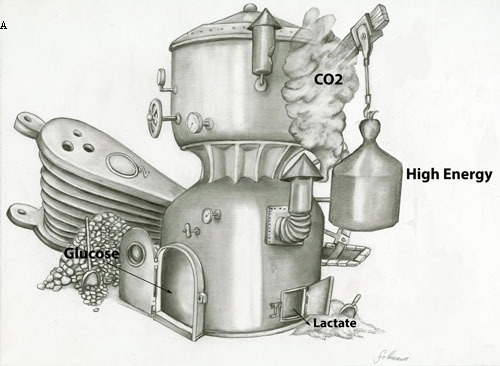

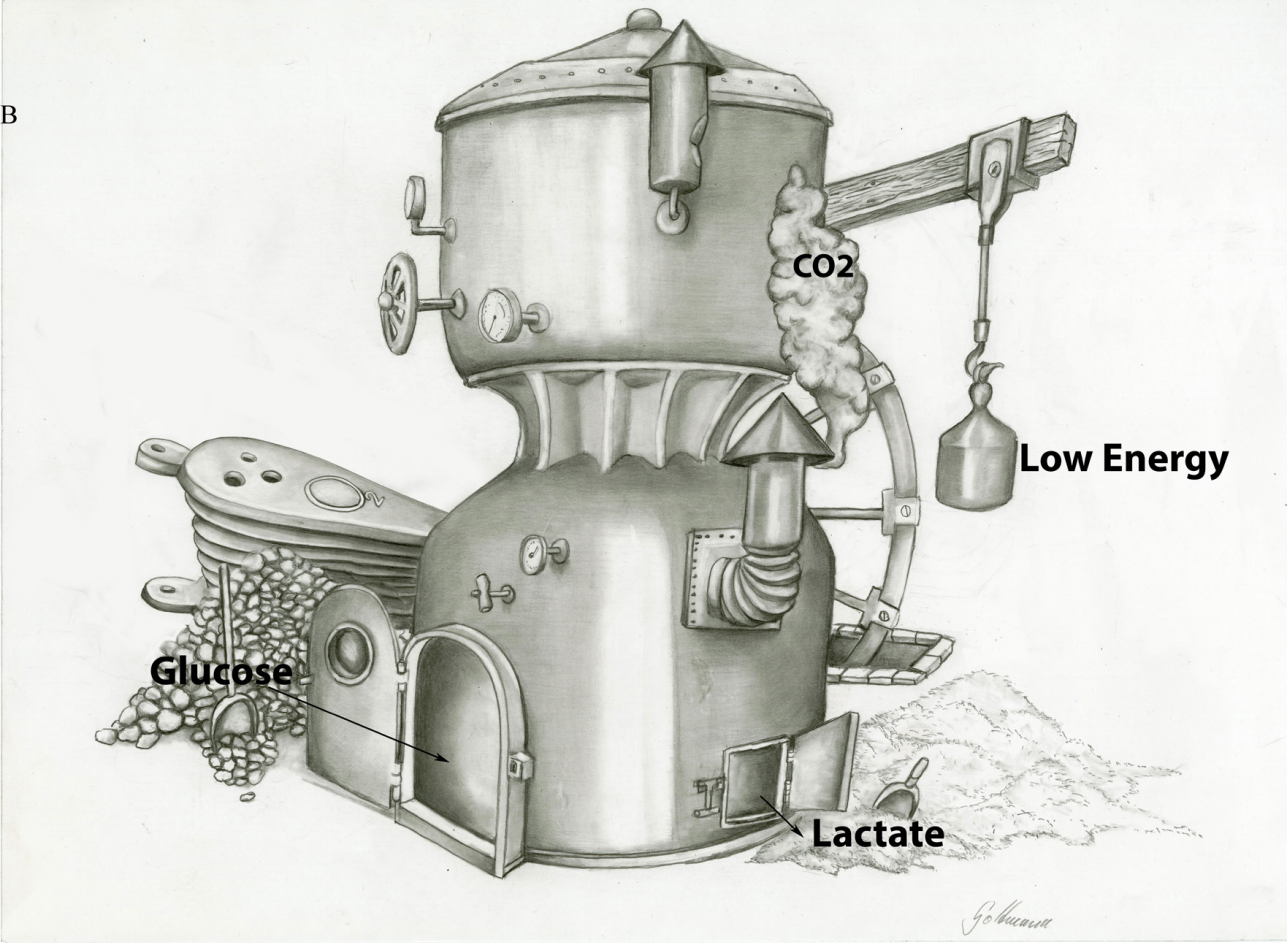

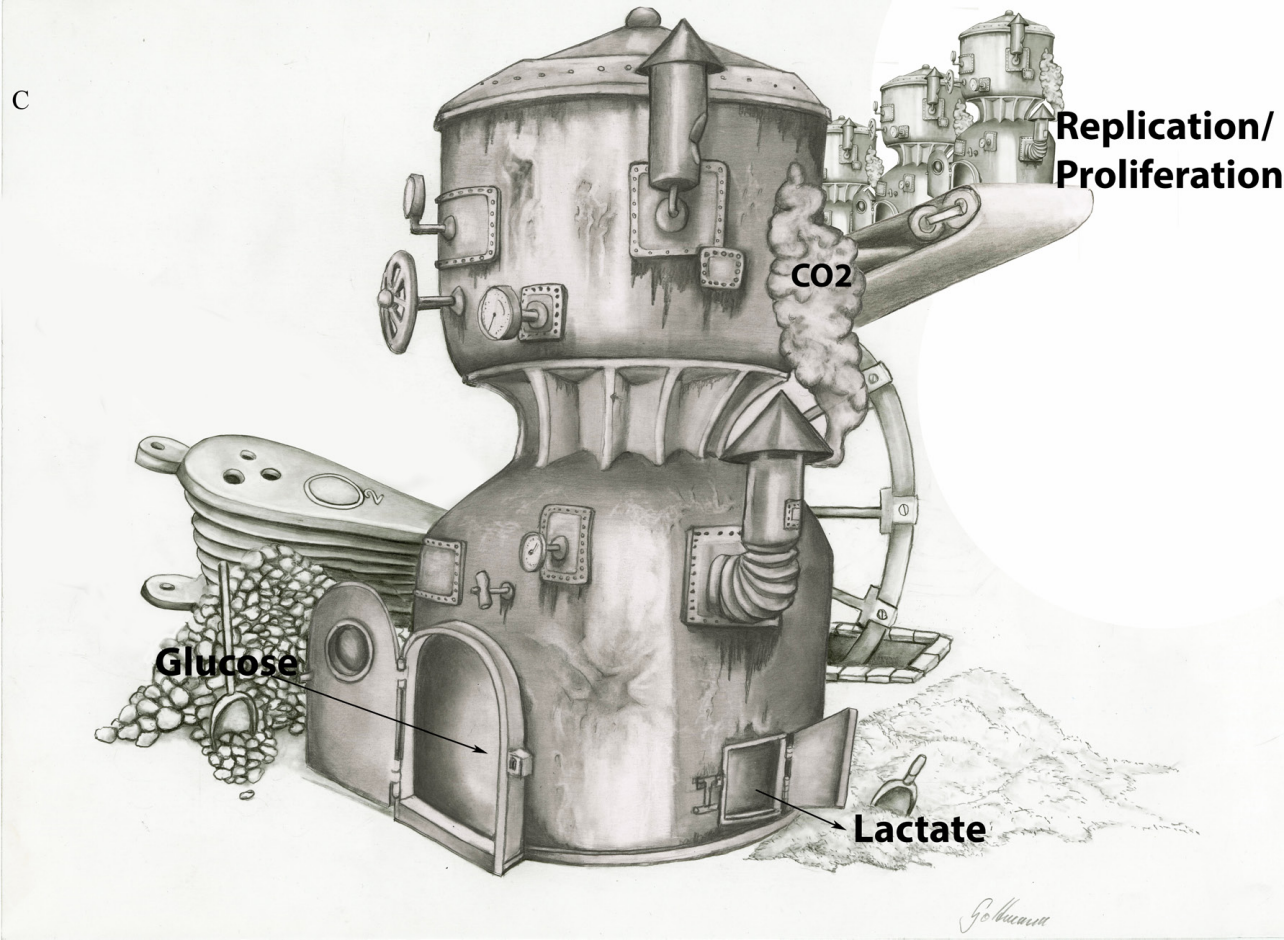
Oxidative phosphorylation in the presence of a) high levels of or b) low levels of oxygen, where glucose is metabolized to H0 and CO with the production of a) high ATP or b) low ATP in a normal cell. c) Proliferating or tumor cells produce low levels of ATP and convert glucose into lactate in the presence of oxygen. This is called aerobic glycolysis or the Warburg effect. It is postulated that tumor cells use the energy for proliferation and replication

Kennedy and Frank [94] investigated human retinal epithelial cells and confirmed the findings of Katavetin et al. [95] who showed that high glucose levels diminished the effect of hypoxia on VEGF. Low glucose and hypoxia (i.e. low pO_2_) resulted in a significant increase in VEGF levels and the authors concluded that the cells used VEGF and angiogenesis as a compensatory mechanism when both fuel sources (oxygen and glucose) were depleted. As clearly shown in Figure 4, tumor cells need a high concentration of glucose, a fact exploited in FDG (F- Fluordesoxyglucose) PET scans.

Tumors will not grow larger than 1-2mm^3^ if neovascularisation, with the formation of an intra-tumor capillary network, does not take place. Neovascularisation seems mainly to be induced by VEGF but recently nitric oxide (NO) has also been implicated in this process [89, 96]. Hypoxic head and neck tumors have a high glucose uptake and the pimonidazole staining for hypoxia shows that the cells are far from blood vessels [3]. Shi et al. [97] implicated VEGF in cell proliferation. It is therefore possible that the induction of VEGF by chronic hypoxia (which occurs far from the blood vessels and is associated with a high glucose uptake) is for cell proliferation and not for angiogenesis whereas acute hypoxia (which is found closer to stunted and/or blind blood vessels) induces VEGF for angiogenesis.

VEGF's role in tumor oxygenation via the vascularisation mechanism remains undisputed and needs to be considered in all assessments of tumor hypoxia.

### Glucose transporter 1 (GLUT-1) and tumor hypoxia

Cells primarily use glucose as an energy source which is converted to H_2_O and CO_2_ to produce ATP in the presence of oxygen (oxidative phosphorylation). In the absence of oxygen, glucose is converted to lactate (anaerobic glycolysis, Figure 4). Otto Warburg and his co-workers in 1920 demonstrated that compared to normal cells, tumor cells have a significant predilection for glucose metabolism under aerobic conditions, coined the Warburg effect [98, 99]. In his focus on glycolysis, Warburg demonstrated a reversed Pasteur effect by the inhibition of oxygen fermentation, implicating the glycolytic production of lactate even in the presence of sufficient oxygen, thus bypassing the entry of pyruvate into the citric acid cycle. The protons diffuse out of the cell into the extracellular environment to maintain intracellular homeostasis [98, 99].

Increased HIF-1alpha is associated with increased glucose consumption in a renal cell carcinoma cell line [100]. More glucose transporters are needed for more glucose uptake, so increased GLUT1, a glucose transporter, correlates with poor outcome for HNSCC patients, since it decreases apoptosis and therefore favors tumor growth [101, 102]. Grimm et al. [101] found that patients with increased GLUT1 and transketolase-like protein 1 (TKTL1) had a worse prognosis compared to patients with low GLUT1 and low TKTL1 levels. Mayer et al. [103] has questioned the antibody used for TKTL1 detection and concluded that TKTL1 does not regulate glucose metabolism in malignant cells. Parallel to lactate production, tumor tissue is acidified, has increased invasiveness and eventually leads to metastasis [104]. The increase in lactate caused by the increased glycolysis could be used as a biomarker in measuring tumor hypoxia.

### Lactate and tumor hypoxia

It seems that any proliferating cell can use aerobic glycolysis [98] and that leukemia cells and lung cancer cells that are in direct contact with the bloodstream are highly glycolytic. Peppicelli et al. [105] hypothesized that increased hypoxia leads to increased glycolysis resulting in increased lactate and therefore increased acidosis which in turn compromises the surrounding cells and results in increased metastasis. This hypothesis was based on the investigations by Brizel et al. [106] and Ziebart et al. [107]. Ziebart et al. [107] took biopsies from 29 HNSCC patients with stage T3 or T4 tumors and from 9 control patients. They showed that lactate was increased and glucose was decreased in tumors compared to normal tissue. Walenta et al. [104] found that high tumor lactate levels, as measured by bioluminescence imaging, correlated to increased incidence of metastasis, tumor recurrence and a decreased overall survival. They found a positive correlation in the distribution patterns of ATP, glucose and lactate which is surprising considering that aerobic glycolysis is known to be an inefficient form of ATP production. Furthermore, lactate was not correlated to tumor hypoxia. Mariappan et al. [108] found a correlation between serum lactate levels and brain tumor grade. Unfortunately, no systemic condition of the patient was taken into account, especially since diabetic patients and chronic alcoholism have a higher basal serum lactate level than non-diabetic patients [109, 110]. No correlation was found between lactate levels and tumor staging [106, 107, 111]. Lactate transporter monocarboxylate transporter 4 (MCT-4) has to be induced by hypoxia and MCT-1 is needed to transport lactate in surrounding tumor cells. MCT-1 expressing cells show expression of KI-67 as well, indicating a higher replication.[112] HPV-positive cell lines appear to metabolise lactate more efficiently than HPV-negative cell lines [113]. Thus far, tumor lactate has been correlated with and seen as a reliable metabolic predictor for poorer prognosis and metastasis but the relationship between tumor hypoxia and lactate is still unclear [114].

### Lactate Dehydrogenase (LDH) and tumor hypoxia

Lactate dehydrogenase catalyzes the reversible conversion of pyruvate to lactate. Lactate dehydrogenases are hetero or homo tetramers encoded by LDHA and LDHB genes. There are five isoenzymes: LDH1, LDH2, LDH3, LDH4 and LDH5. LDH5 expression independently correlates with poor prognosis and metastasis in HNSCC and is not expressed in normal head and neck mucosa [115]. Oropharyngeal tumors have higher LDH5 expression than other head and neck tumors. The serum LDH level in HNSCC patients is an independent prognostic factor and Hsieh et al. [116] and Yao et al. [117] found that serum LDH above the upper limit of normal correlates with poor survival. LDH activity in saliva has also been correlated with periodontal disease [118]. LDH expression is significantly increased (88 %) in saliva from oral squamous cell cancer patients compared to age and sex-matched controls, independent of smoking, alcohol consumption and other diseases [119]. On the other hand an inverse correlation between tumor grade and LDH activity has been shown, furthermore HNSCC tumor cell lines xenografted into mice showed a correlation between LDH and pimonidazole staining [120, 121]. Although increased serum LDH is mainly correlated with the incidence of cancer, its expression increases drastically during infection [122]. A link between both serum and tumor LDH and tumor hypoxia and prognosis is well established and should be included in future investigations.

### CA-IX (Carbonic Anhydrase IX) and tumor hypoxia

CA-IX comes from a family of zinc metalloenzymes that catalyze the reversible hydration of carbon dioxide. It is a transmembrane protein involved in various biological processes such as acid-base balance, the formation of saliva, cerebrospinal fluid and gastric acid and may be involved in cell proliferation. CA-IX is a prognostic marker for a number of malignancies [123]. In cervical cancer [124] and in HNSCC patients [25] there is no correlation between CA-IX staining and pO2 values. This is not supported by all research as Beasley et al. [125] confirmed the hypoxia dependent induction of CA-IX in three HNSCC cell lines.

A meta-analysis including 16 studies and 1470 patients showed that CA-IX is correlated with poor survival independent of tumor hypoxia [126]. The meta-analysis did not take into consideration the different locations of the HNSCCs, only tumor size, tumor grade and nodal status. Brockton et al. [127] analysed 91 biopsy samples from HNSCC patients who had radio- and chemotherapy and found that CA-IX was not correlated to smoking status, gender, age, tumor site, tumor stage, performance status or response to therapy. They did, however, find a correlation between high stromal CA-IX levels and HPV p16-negative status and poor survival. In a cohort of 61 HNSCC patients with mainly HPV p16-negative tumors, there was no correlation between CA-IX and gender, age, tumor site, tumor stage, performance status or response to therapy but a correlation between CA-IX and smoking status and between high stromal CA-IX and poor survival existed [128]. So, there seems to be prognostic relevance with a high stromal expression of CA-IX, however, our assessment may not be reliable or sensitive enough to verify its clinical relevance in tumor hypoxia.

### Osteopontin and tumor hypoxia

Osteopontin (OPN) is a phosphoglycoprotein that plays a role in bone remodeling, immune response and inflammation [129]. Le et al. [130] and Nordsmark et al. [25] were able to show an inverse relationship between plasma osteopontin and pO_2_ in HNC patients but no correlation between tumor osteopontin (obtained through immunohistochemical staining) and pO_2_ levels. A year earlier, Bache et al [131] found a correlation between osteopontin immunoreactivity, HIF-1alpha, Hb and VEGF but not with pO_2_ or CA-IX. They also found no correlation between HIF-1alpha, CA-IX and pO_2_. Overgaard et al. [132] used data from 320 patients from the Danish Head and Neck Cancer Group (DAHANCA) and found that high concentrations of plasma osteopontin predicted poor outcome but this could be attenuated when the patients were treated with nimorazole, a hypoxia radiosensitizer, during radiotherapy. Lim et al. [133] analysed data from 578 HNSCC patients (Trans Tasman Radiation Oncology Group: TROG study) and found no correlation between OPN and poor survival nor did they find any effect with the tumor radiosensitizer tirapazamine. It is not clear why these results are so different to those found in the DAHANCA study, though the TROG study appeared to be more stringent in their analysis, and the blood samples used in the DAHANCA study had been stored for nearly 19 years, while those used in the TROG study were fresh. Osteopontin secretion furthermore showed no correlation with hypoxia in 4 nasopharyngeal cancer cell lines [134]. Plasma osteopontin levels in metastatic nasopharyngeal carcinoma patients and HNSCC patients were elevated compared to controls and were a significant predictor of response to radiotherapy [134]. Tumor hypoxia increases metastasis and Courter et al. [129] found that osteopontin regulates tumor growth and metastasis by inhibiting apoptosis.

Low-grade inflammation as is evident in diabetes and metabolic syndrome has been associated with osteopontin derived from macrophages. Ahmad et al. [135] found a positive correlation between circulating osteopontin, fasting blood glucose and BMI. Smoking increases plasma osteopontin in type 2 diabetic patients [136]. The evidence correlating both serum and tumor osteopontin with tumor hypoxia and survival is mostly supportive, however some contradictory evidence mandates further research-

### Nitric Oxide and tumor hypoxia

Nitric oxide is a free radical and an important signaling molecule involved in vasodilation and in protection against ischemic damage. Both salivary and serum nitric oxide can be used to determine oral mucosal disease, as well as oral precancer and cancer status in patients [137-140]. NO is synthesized by nitric oxide synthase of which three isoforms are known, namely, inducible (iNOS), neuronal (nNOS) and endothelial (eNOS) nitric oxide synthase. It appears that the activity of VEGF may be upregulated by NO generation by eNOS and thus be implicated in tumor growth[96]. iNOS has also been shown to be a HIF target [141]. The role of NO in neovascularisation has already been discussed.

Despite significant complexities, current evidence supports NO as a prognostic marker and it has been shown to induce non-oxygen dependent HIF stimulation. Although increased NO is linked to poor outcome in cancer patients, NO has been shown to significantly increase cell apoptosis via p53 expression [142].

### p53 and tumor hypoxia

p53 is a tumor suppressor protein encoded by the TP53 gene in humans. It is activated by a myriad of stressors, one of which is oxidative stress. In a mouse model DM2, a protein encoded by the DM2 gene inhibits p53 by transporting it from the nucleus to the cytosol or by attaching ubiquitin to p53 so that it can be degraded. Adduri et al. [143] found that p53 nuclear stabilization differed between ‘old’ and ‘young’ patients with squamous cell carcinoma of the tongue, being lower in ‘older’ patients. Both p53 nuclear stabilization and p53 mutations were correlated with poor survival in the patients. Persistent expression of wild type p53 can be seen as a positive predictor for radiosensitivity [144]. This is in contrast to Portugal et al. [145] who found no correlation between p53 gene mutation and recurrence or survival status but who did, however, find a correlation between p53 gene mutation and alcohol use. The correlation of p53 gene mutation and alcohol-use corroborated the results of Sorensen et al. [146] who found that non-smoking, non-drinking young patients with squamous cell carcinoma of the tongue had less p53 mutations than their counterparts. As mentioned earlier, alcohol and cigarette use are independent prognostic factors for poor survival.

Thus far it has been thought that inhibiting the MDM2/HIF-1alpha interaction would result in tumor cell death in hypoxia [147] but recently Joshi et al. [84] found that MDM2 causes HIF-1alpha to be degraded during hypoxia so inhibiting the MDM2/HIF-1alpha interaction may actually result in tumor growth, not death. The role of p53 and its mutations can be regarded as well established in HNSCC, however its precise interrelationship with tumor hypoxia needs to be explored and clarified further.

Genetic regulation and interplay in cancer is demonstrated by the extensive research on microRNAs.

### MicroRNAs and tumor hypoxia

MicroRNA (miRNAs) are short noncoding RNAs that posttranscriptionally regulate target messenger RNAs [148]. 7 microRNAs are consistently upregulated in HNSCC, namely, miR-21, miR-7, miR-155, miR-130b, miR-223, miR-34b, miR-210 and 4 are consistently downregulated, namely, miR-100, miR-99a, miR-125b, and miR-375 [149]. We will mainly take a closer look at miR-21, miR-210 and miR-375.

miR-21 which is the most researched miRNA, is upregulated in various cancers [150]. It is anti-apoptotic and is expressed in the tumor stroma of HNSCC tumors [148]. Gee et al. [15] could find no correlation between miR-21 and other markers for tumor hypoxia in HNSCC patients. *In vitro*, Polytarchou et al. [151] found that Akt2 promotes hypoxia resistance via upregulated miR-21 whereas Loayza-Puch [152] found that hypoxia downregulates RECK (a tumor-suppressor protein) via upregulated miR-21.

miR-210 is consistently induced by hypoxia as a HIF target in normal and transformed cells [153]. It correlates with markers of tumor hypoxia in HNSCC patients, such as HIF-1alpha, CA-IX and a 99-gene hypoxia metagene [15]. To our knowledge, miR-210 is only regulated by hypoxia.

miR-375 is downregulated in HNSCC alluding to it being a tumor suppressor. Possible target genes of miR-375 are those involved in cell growth and insulin secretion [154]. Congruent with its effect on insulin secretion, high levels of miR-375 correlate with the incidence of diabetes and miR-375 has been suggested as a biomarker for not only cancer [155, 156] but Type 2 diabetes as well [157]. Overexpressing miR-375 *in vitro* in laryngeal cancer cell lines (SNU-48 and SNU-899) resulted in increased apoptosis and decreased proliferation and invasivity [158] which fits with the evidence that miR-375 acts via phosphoinositide-dependent protein kinase-1 [159]. miR-375 correlates to tumor staging and tumor size but no experiments were done regarding tumor hypoxia directly [160].

Although miRNAs look promising as biomarkers for HNSCC, they are regulated by various cancers and diseases and their role in tumor hypoxia needs further elucidation. Table 1 and Table 2 depict a further list of possible inflammatory and genetic biomarkers relevant to HNC as found in the current literature.

**Table 1:** Inflammatory biomarkers related to head and neck tumors and tumor hypoxia

Biomarker	Material tested	Method	Result interpretation	Reference
CD8 (Tumor Infiltrating Lymphocytes)	tumor	*IHC	High CD8+TIL (HPV positive tonsillar and base of tongue SCC) = better survival	[179]
CD44 (Tumor Infiltrating Lymphocytes)	tumor	IHC	Low expression (HPV positive oropharyngeal SCC) = very high survival	[180]
IDO (Indoleamine 2,3-dioxygenase)	tumor	IHC	High tumoral (laryngeal SCC) expression = poor outcome (inhibited local immunity)	[181]
PD1 (programmed death 1)	tumor	IHC	High expression (HPVpos.HNSCC) = good prognosis. It can be efficiently blocked by anti-PD1 (melanoma, colorectal and renal cancer)	[182]
MIF (Macrophage Inhibitory Factor)	tumor	IHC	Increased expression of MIF in tumor cells & TILs predicts improved patient survival (nasopharyngeal carcinoma)	[183]
IL-15 (Interleukin)	tumor	IHC	High intratumoral expression = poor clinical outcome (HNSCC)	[184]
TGFβ1 (Transforming growth factor β 1)	tumor	IHC	Overexpression might predict oral cancer metastasis	[185]
SMAD 6 & 7 (regulators of TGFβ pathway)	tumor	IHC	Loss of expression = poor prognosis (HNSCC)	[186]
CSF1R (Colony stimulating factor 1 receptor)	tumor	IHC	Up-regulated in radiation-resistant HNSCC	[187]
MALT1 (Mucosa-associated lymphoid tissue 1)	tumor	IHC	Loss of expression = poor prognosis (oral cancer)	[188]
CXCR4, also known as SDF-1	tumor	IHC	High expression = poor prognosis (tongue cancer)	[189]
ArginaseII	tumor	IHC	Absence of expression = prolonged overall survival (HNSCC)	[190]

* IHC: Immunohistochemistry

**Table 2:** Genetic biomarkers related to head and neck tumors and tumor hypoxia

Biomarker	Material tested	Method	Result interpretation	Reference
PTEN (Phosphatase and Tensin homolog)	tumor	*IHC	High expression in HNSCC = significant gain in loco-regional control	[191]
mTOR (mechanistic Target of Rapamycin)	tumor	IHC	High expression = shorter DFS (laryngeal cancer)	[192]
PK-M2 & PK-M1 (Pyruvate kinase isozymes M1/M2)	tumor	IHC	Isoform switch to higher expression of PK-M2 = poor prognosis (HNSCC)	[193]
integrin αvβ5	tumor	IHC	High expression = high risk of metastasis (Laryngeal SCC)	[194]
PDK-1 (Pyruvate dehydrogenase kinase-1)	tumor	IHC	High expression = poor prognosis (HNSCC)	[195]
Ki-67	tumor	IHC	High expression = good prognosis (Oral SCC)	[196]
p27	tumor	IHC	High expression = good response of HNSCC to chemotherapy (Cisplatin+5FU)	[197]
EGFR (epidermal growth factor receptor)	tumor	IHC	High expression predicts better loco-reg. control of HNSCC with Contin. Hyperfract. Acceler. Radioth. (CHART)	[198]
LOX (Lysyl oxidase)	tumor	IHC	High expression = poor overall survival (HNSCC)	[199]
*DSPP, OPN, MMP-9	tumor	IHC	Expression in negative tumor margins predict recurrence of oral cancer	[200]
MMP-13 (Matrix metalloproteinase-13)	tumor	IHC	high nuclear MMP-13 expression = poor outcome (tongue cancer)	[201]
CytK13, CytK 14 and CytK 16 (Cytokeratins)	tumor	IHC	Loss or downregulation = poor prognosis (recurrence & metastasis of tongue cancer)	[202]
CytK19 (Cytokeratin 19)	tumor	IHC	Decreased in tumor: metastasis (HNSCC)	[203]
Slug (necessary for HIF-1α induced cadherin switch)	tumor	IHC	High expression = tumor invasion and short survival (HNSCC)	[204]
HDAC2 & pVHL (Histone deacetylase 2 & von Hippel–Lindau protein)	tumor	IHC	High HDAC2 & low VHL expression = advanced stage and poor prognosis (Oral SCC)	[205, 206]
CTGF (Connective Tissue Growth Factor)	tumor	IHC	High expression = poor prognosis (HNSCC)	[207]
Galectin 1 and 3	tumor	IHC	High expression correlates with tongue SCC metastasis	[208]

* IHC: Immunohistochemistry; DSPP: dentinsialophosphoprotein; OPN: osteopontin; MMP-9: matrix metalloproteinase-9 [70]

### Genetic profiling

Various efforts have been made to define and utilize genetic head and neck tumor profiling. A number of gene expression subtypes can be identified nanely basal (31%), mesenchymal (27%), atypical (24%) and classical (18%). Some promising findings have been forthcoming and further research is needed to elucidate their potential clinical relevance.

For example, it was shown that the *NFE2L2* oxidative stress pathway is a tobacco-related signature not specific to an anatomic tumor site. HPV-positive tumors show recurrent deletions and mutations of TNF receptor associated factor 3 gene loss as well as mutations in exon 9 of the PIK3CA helicase domain. Tumor cause can now thus be confirmed genetically [60]. A 26 gene hypoxia signature has been used used to calculate a hypoxia score to predict treatment benefit for a hypoxia adapted treatment with carbogen and nicotinamide protocol before radiotherapy in 157 larynx cancer patients. High expression of hypoxia related genes did predict for a poorer 5 year survival however not for the value of hypoxia modification treatment [161]. In an attempt so simplify further, clinically relevant research, smaller, more robust signatures or metagenes have been developed [162, 163].

Tourstup K et al. [155, 156] developed a 15 gene hypoxia classifier with prognostic impact that was verified it in the DAHANCA 5 study cohort and is now being implemented in a prospective accelerated chemo-radiotherapy trial with or without the radiosensitizer nimorazole in HPV negative HNSCC patients (EORTC 1219).

### Imaging

Imaging may be a possible alternative to profiling tumor hypoxia. [F-18] fluoromisoni-dazole (FMISO), (18)F-fluoroazomycin arabinoside (FAZA)-PET, dynamic contrast enhanced MRI, arterial spin labelling (ASL) MRI and other MRI techniques have all shown promise in this regard, and may allow hypoxic tumors to be defined in a non- invasive and possibly continuous manner. Imaging may even allow us to monitor treatment response [164-168]. Near infra-red spectrometry may be a solution to predict oxygen content in more superficial tumors [169]. The major drawback of imaging thus far has been the lack of research proven correlation of the imaging to the various hypoxia parameters.

## DISCUSSION

HNC comprises many different cancers in terms of aetiology, localization, duration, size, metastatic potential etc., yet in many analyses these diverse malignancies are often grouped together that could lead to erroneous conclusions. It may therefore be more prudent to deal with each cancer separately and define the mechanisms and treatment separately, then again this further subtyping will further much needed diminish statistical power [89, 170-172]. As a common denominator all these cancers are linked by tumor hypoxia as a prognostic factor, however the molecular pathways involved may be different and therefore treatments need to be more individualized. As described tumor hypoxia is only universal in terminology, but extremely diverse in term of definition as the measuring parameters range from physical measurement to various up and downstream metabolic products.

HIF-1alpha, CA-IX and Glut1 are used as the main markers to represent tumor hypoxia, but they fall short of the criteria for a true endogenous marker as listed by Janssen et al. [3]. These markers can, however, potentially be used to predict disease progression or overall survival, but independently of hypoxia as especially HIF is also influenced by non hypoxic driven factors. Imaging techniques such as FMISO, FAZA-PET and various MRI techniques such as arterial spin labeling, BOLD-MRI and quantitative diffusion MRI will probably lead the way to non invasive hypoxia measurements, but the metabolic interpretation will have to be validated with the various biomarkers as discussed [167, 173-175].

HPV, with HPV E7 being a known HIF-1alpha protein interactor [54], is becoming one of the main risk factors for HNSCC. Specifically oropharyngeal cancer with smoking acting as an compounding risk factor with the risk of death increasing steadily with every additional packyear [176]. Even though HPV-infected cancers are aggressive and grow rapidly, prognosis is generally good whereas tumor hypoxia in both HPV-positive and negative tumors results in poorer prognosis [177]. Does the answer to the differences in outcome lie in individualised immune response? Inflammation is known to play a role in tumor hypoxia but the role of inflammation in HPV-positive cancer is still unclear. Can the differences be attributed to the difference in the miRNA profile as suggested by Lajer et al. [178]?

There is no real standardization between clinical trials in terms of parameters set, e.g. the hemoglobin cut-off to determine anemia varies, pO_2_ as parameter for hypoxia varies and the term tumor hypoxia is erroneously used as a collective term for what should be called tumor oxygenation. It is therefore, surely not sufficient to look at a single marker for hypoxia but rather one probably needs several as shown by Hsieh et al. [116].

Despite the above outlined limitations in some of the understandings of tumor hypoxia, there are centres implementing adapted radiation protocols as standard of care [24, 25].

As genetic profiling develops, the intricacies of the complex tumor hypoxia drama may be unraveled further, however it may also deepen the plot with hundreds of new key suspects arising, each playing a supporting role to the main role player, oxygen. Despite the ever expanding complex plot, there have been some positive discoveries with potential therapeutic relevance, like HIF-1alpha. This will pave the way for more individualized treatment, however, much more needs to be uncovered to be able to routinely follow and adapt treatment response.
